# Artificial Intelligence to Reduce Ocular Health Disparities: Moving From Concept to Implementation

**DOI:** 10.1167/tvst.10.3.19

**Published:** 2021-03-18

**Authors:** John P. Campbell, Ciku Mathenge, Hunter Cherwek, Konstantinos Balaskas, Louis R. Pasquale, Pearse A. Keane, Michael F. Chiang, on behalf of the American Academy of Ophthalmology Task Force on Artificial Intelligence

**Affiliations:** 1 Department of Ophthalmology, Oregon Health & Science University, Portland, OR, USA; 2 Rwanda International Institute of Ophthalmology, Kigali, Rwanda; 3 Orbis International, New York, NY, USA; 4 Institute of Ophthalmology, University College London, London, UK; 5 Medical Retina Service, Moorfields Eye Hospital NHS Foundation Trust, London, UK; 6 Eye and Vision Research Institute, New York Eye and Ear Infirmary at Mount Sinai, Icahn School of Medicine at Mount Sinai, New York, NY, USA; 7 National Eye Institute, National Institute of Health, Bethesda, MD

**Keywords:** artificial intelligence, global health, implementation science, retinopathy of prematurity, age-related macular degeneration

## Introduction

An estimated 30 to 40 million people are blind worldwide from preventable or treatable conditions because the system into which they were born failed to detect and/or treat their disease.^1^^,^^2^ The reasons for this are complex. Geographic inequalities are observed both between and within countries. On the macro level, the majority of blind people in the world live in low-income countries; on the micro level, geographic inequalities are often observed, with higher rates of blindness in rural and underserved regions, even in high-income countries.^1^^,^^2^ Further, the relationship between economic development and blindness is more complicated than simply access to health care because some diseases, such as trachoma and onchocerciasis, are directly related to poverty and have become less common with economic development. Other diseases become more common with economic development, such as diabetic retinopathy, and with longer lifespan, such as age-related macular degeneration (AMD), glaucoma, and cataract. However, in each of these conditions, inequitable access to eye care contributes to a higher prevalence of blindness in low income, medically underserved, and rural communities around the world.

The emergence of artificial intelligence (AI) in medicine has raised hopes that this technology, which has demonstrated the ability to make medical diagnoses from images, might reduce inequalities in access to ophthalmologic diagnosis and ultimately care.^3^ Although the applications of AI in ophthalmology are myriad, the motivating use case here would be for secondary prevention of blindness as a force multiplier to enable ophthalmologist-level diagnosis to reach places where health systems cannot meet the clinical need using existing resources. A secondary goal might be to improve the efficiency of care delivery in both high- and low-income regions. One method to achieve this would be to distribute the physical location of imaging systems in a spoke-and-hub model to primary health clinics or local optometric clinics, with only positive exams being referred for in-person care. By reducing the screening burden on clinicians for patients not requiring treatment, resources in both low- and high-income regions might be better utilized to maximize the population level impact of existing clinicians. Additionally, in the COVID-19 era, there has been renewed attention on modes of care delivery that utilize telehealth to minimize unnecessary visits to healthcare facilities.^3^^,^^4^

The hypothesis that AI might lead to improved population-level outcomes is predicated on the assumption that the limiting factor is detection, rather than treatment, of disease. However, screening for conditions such as blinding cataract can be done easily by community health workers without deployment of AI. In regions where systems exist to transport patients efficiently to providers (or vice versa) for treatment, this model of primary health worker-driven remote screening can work as part of a comprehensive care delivery model with a population-level impact on blindness reduction. In India, for example, a national focus on cataract blindness and the implementation of multiple innovative care-delivery models have resulted in an improved cataract surgical rate (number of cataract surgeries per population) and dramatically reduced the prevalence of blindness over the last 30 years.^5^ In other cases, identification of diseases such as diabetic retinopathy requires more than the skill-set of a community health worker; thus, diagnosis of the retinal disease remains a key limiting step. This is one area where telemedicine has had a beneficial effect using remote interpretation of digital fundus images. There are a number of examples of successful implementation of telemedicine programs for diabetic retinopathy that have led to reduced incidence of blindness at the population level.^6^^–^^8^

The fact that both cataract and diabetic retinopathy remain leading causes of blindness should therefore lend some humility to any predictions regarding the ability of technological solutions, such as telemedicine or AI, to measurably impact the problem of global blindness. This is reflective of a more general truth at the intersection of clinical and public health ophthalmology—that is, the low-hanging fruit of blindness prevention and treatment is the implementation of existing knowledge.^2^ We know how to treat cataracts and improve vision, we know how to screen for diabetic retinopathy and reduce the incidence of blindness, and we know how to minimize vision loss from neovascular AMD with consistent treatment; yet, in each case we have failed to deliver the best possible care to the greatest number of people, even in well-developed health systems, and especially in medically underserved populations worldwide.

This is the realm of dissemination and implementation science,^9^^–^^11^ which focuses on the barriers that prevent proven interventions from widespread utilization and maximal population impact. In this paper, we outline a framework from implementation science to consider the potential impact of AI on addressing unmet needs in the challenge to reduce health disparities and reduce blindness worldwide. In doing so, it is worth considering the potential added value of AI compared to existing solutions, such as telemedicine, to overcome practical implementation challenges that have prevented existing solutions from achieving impact at scale.

## Implementation Science

Implementation science is the study of how, when, why, and to what extent evidence-based healthcare interventions are incorporated in a population.^9^^–^^11^ It provides evidence on whether and how interventions are not just effective but also usable, useful, cost efficient, and scalable. Whereas the gold standard for a therapeutic intervention is a randomized controlled trial, the field of dissemination and implementation science evaluates the translation of “bench to bedside,” or, in the case of AI, “from code to clinic.” For AI to be adopted and used, digital tools must fit seamlessly into people's lives—both members of the public and health professionals. The adoption of any new tool requires a change in practices. The necessary changes must be planned and be acceptable to the relevant user groups. A further consideration is whether an intervention is scalable. This typically involves consideration of local adaptations across care delivery settings, economies of scale, and sustainability over time. To optimize future implementations of AI devices, it will be important to understand factors and processes that influence this implementation. It has been estimated that it takes an average of 17 years from research evidence of benefit to change clinical practice, and that is assuming the healthcare infrastructure supports the intervention.^12^ In that sense, the question before us is perhaps the most challenging application of implementation science: the implementation of novel innovations in the most medically underserved populations where basic healthcare infrastructure may not exist.^11^^–^^13^

The paradigm that we will consider in evaluating the potential of AI devices to impact eye care at scale is that clinical and/or technological innovations, whether a new drug or an AI algorithm, cannot impact public health if they are not adopted by the community and thus available to patients. There have been numerous papers published demonstrating the efficacy of AI to perform a diverse array of image-recognition tasks as well as clinicians perform those tasks, such as the detection of referable diabetic retinopathy, and even some things that clinicians cannot do, such as predict age, gender, and smoking status.^14^ However, to understand what it will take for AI to really impact the problem of preventable blindness, validation needs to reach beyond efficacy in research studies, to effectiveness in real-world populations in specific use-case settings, and finally to understanding the environmental factors necessary for the technology to be maximally impactful in a population. And, it has to add some value compared to existing systems. Simply, we need to consider not just the technology but also how the technology is implemented.

One conceptual model we find helpful is the following: efficacy (performance in a controlled clinical setting) → effectiveness (performance and outcomes in intended population) → implementation (overcoming obstacles to use) → environment (ensuring success through developing sustainable systems), as seen in the Figure.^14^^,^^15^ That is, when a technology has demonstrated efficacy in the preclinical or clinical trial setting, it must demonstrate effectiveness in clinical practice, barriers to adoption must be overcome, and, finally, sustainable systems must be developed to ensure long-term adoption of the innovation. Because evaluation of the efficacy of AI systems is beyond the scope of this paper, we direct the reader to one of several excellent references on this topic.^3^^,^^14^^,^^16^ In this paper, we focus on the rest of the implementation science framework.

**Figure.**
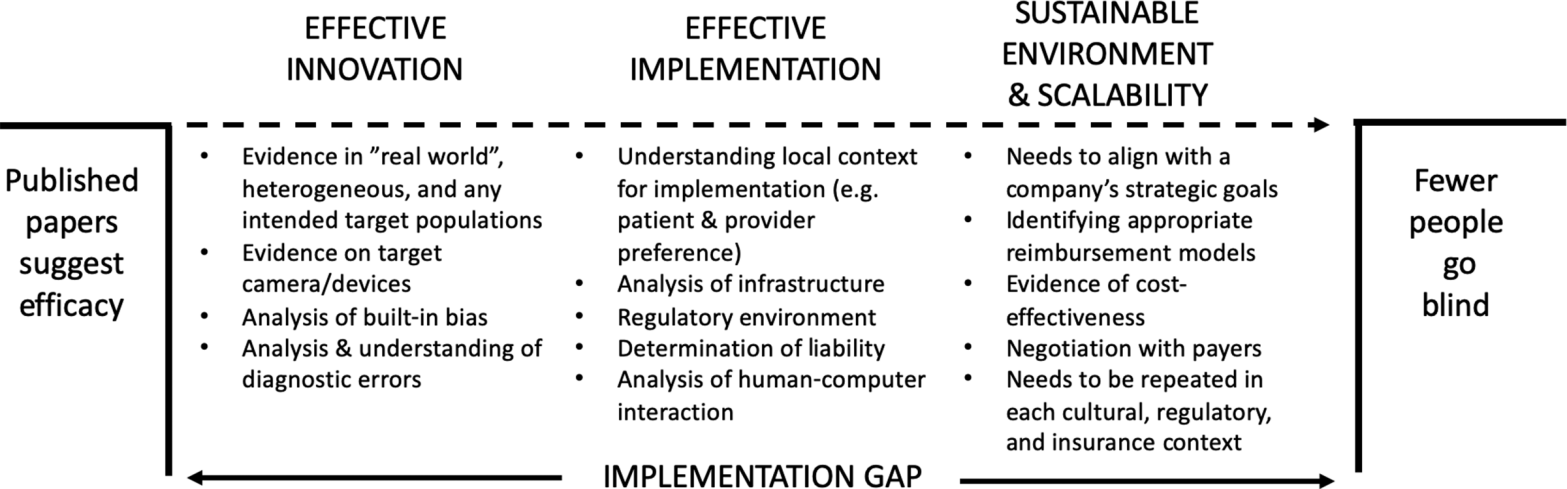
Conceptual model for implementation science. This model simplifies and summarizes one way to look at the steps involved to translate the potential promise of AI into the eventual outcome of reducing human disease.

### Effective Innovations

There is a general principle in clinical science that performance in a controlled clinical experiment does not always translate to similar performance or improved outcomes in practice. The loss of performance increases as the target population becomes more heterogeneous. In the case of AI, datasets used to develop algorithms often differ from datasets in the real world in ways that can substantially affect outcomes, including image quality; patient age, ethnicity, and other demographic factors; prevalence of disease; and image acquisition techniques or cameras.^17^ Moreover, in the same way that clinicians can be biased by factors such as gender or race, AI algorithms can develop biased models, as well, based on pattern association in the training data.^17^ In the spirit of implementing technology to reduce health disparities and follow the principles of justice and nonmaleficence, this will be a key area to explore and address ways to minimize potential harms.

The problem of lower effectiveness is a key potential implementation barrier to the use of AI systems in low-income countries, where many of these variables differ from the original population used for AI training.^19^ We need to encourage further evaluation of AI systems in these populations and not just assume the performance in a clinical research setting will translate to improved outcomes in these new populations. Furthermore, because AI algorithms may miss cases differently than clinicians (for example, only in certain subsets), careful attention must be paid to the pattern of error. In addition, it is critical to pay attention to the sensitivity and specificity of various operating thresholds and the resultant implications of positive and negative tests on the cost-effectiveness of the care. For example, low specificity may result in many false positives, which may lead to added expense, burden to the health system, and potential morbidity. In cases where the effectiveness is less than optimal, one option is to develop de novo algorithms in each population; however, this is often not practically achievable. Alternatively, transfer learning can be utilized to fine-tune an existing model using a smaller subset of data from the intended population. Whereas AI performance is often evaluated in terms of the area under the receiver operating characteristic curve, or overall accuracy, much more is important when evaluating the effectiveness of an AI device.^19^^,^^20^ In a well-known example, computer-aided detection of breast cancer via mammograms worked well in controlled experiments compared to radiologists but led to more biopsies, higher cost, and no increase in detection rates of cancer in clinical practice.^19^^,^^21^

### Effective Implementation

When acceptable clinical performance has been demonstrated in the intended population, specific barriers to clinical adoption must be identified and strategies to overcome those barriers must be evaluated. This may involve qualitative research methods (patient preferences, practitioner preferences, and identification of available technical infrastructure, including hardware and internet access, as well as needs assessment of the training gap, human–computer interaction assessment, risk acceptance, medicolegal framework, and liability). For AI, there are very few technologies that have made it to clinical adoption and demonstrated effectiveness; therefore, the implementation barriers remain largely theoretical.^18^ Nonetheless, the implementation of telemedicine, or lack thereof (pre-COVID-19), for eye care services reminds us of the challenge of taking concepts and technologies that are useful in silico and changing practice. Detailed investigation into the challenges of telemedicine implementation (i.e., whether it was the lack of patient or provider adoption, the lack of favorable financial environment, or the capital expenses required, or all of the above) is beyond the scope of this paper. Nonetheless, it is useful to consider if and how AI devices may add value in a way that traditional telemedicine services did not.

### Sustainable Environment (Scalability)

Even after effectiveness has been demonstrated and the community wants to adopt the technology, there should be a sustainable strategy for the technology to be put into practice, scaled to need, and maintained and updated long-term. This requires more than a compelling cost-effectiveness argument; it requires the incentives of the innovators, manufacturers, payers, and recipients of the technology to be aligned. In the case of autonomous AI for diabetic retinopathy screening, for example, the technology preceded the financial model for successful implementation in the United States, and it remains to be seen whether the business model will change the existing paradigm for screening on a large scale or reduce population prevalence of blindness from diabetes. Complicating matters in LMIC, both the implementation barriers and the environmental factors to ensure financial sustainability will vary by region and regulatory authority, requiring some combination of “out of the box” technology development with local customizable problem solving to overcome regulatory, infrastructure, and healthcare system barriers.

## High-Value Targets in Ophthalmology for AI-Based Screening

As discussed above, the main potential uses for AI would be detection of disease that requires (1) some clinical expertise and or technology to diagnose, (2) a predictable disease course where the disease can be identified early, and (3) treatment is available and improves outcomes. In terms of the ophthalmic diseases that are highly prevalent worldwide, several potential target diseases seem attractive for AI device innovation.

### Refractive Error

In blindness surveys, refractive error is often the most common causes of vision impairment and blindness around the world, and so “easily” treatable that ophthalmologists forget its day-to-day practical impact for millions.^1^^,^^22^ But, refractive error provides an excellent case in point for the importance of implementation research, as its diagnosis requires some technical skill, the patient must be able to afford the treatment, and the supply chain and market must support distributors of eyeglasses. In terms of diagnosis, several low-cost autorefractors are available but not widely used in the United States for regulatory reasons. Recently, a number of groups have demonstrated proof of principle that refractive error, like diabetic retinopathy, can be diagnosed from fundus photographs using deep learning, providing one more way to automate the detection of this disease.^23^^–^^25^

As myopia continues to be a leading and growing cause of visual impairment globally, it remains to be seen how this proof of efficacy might translate into effective and scalable systems of care and how AI might play a role in the care pathway.^26^ One particularly intriguing case of rapidly scaling technology is the smartphone-based mobile refractometer available via GoCheck Kids.^27^^,^^28^ This cloud-base service provides point-of-care diagnosis of refractive error for use in the primary care setting and has created a data-rich environment that has now used AI to improve end-user actions to capture the best image possible to improve the diagnostic accuracy. That is, data are being used to improve not only the accuracy of algorithms but also the quality of end-user input. This highlights two potential advantages of AI or otherwise efficacious low-cost mobile health devices over telemedicine: the potentially low-cost barrier to entry (no expensive capital equipment) and real-time diagnosis (no opportunity cost for the eye-care provider and no delay in feedback to the screener or patient).

### Diabetic Retinopathy

Diabetic retinopathy (DR) is one of the leading causes of permanent blindness in the working-age population, affecting roughly a quarter of the more than 400 million people with diabetes worldwide.^29^ The epidemiology of DR has changed over time, with the highest worldwide prevalence now in the Western Pacific Islands and the highest increase in Sub-Saharan Africa. Even though screening and early detection have been proven to prevent blindness from DR, the prevalence has been increasing due to changes in the underlying populations at risk and inadequate screening. The DR screening model is a quintessential use case for AI in that it has traditionally required anyone at risk to be seen by an eye-care provider (ophthalmologist or optometrist) for a dilated ophthalmoscopic examination. Ignoring, for the moment, the fact that the sensitivity and specificity of an eye exam are far from ideal, this system is inefficient and expensive for both the patient and the provider, especially because the vast majority of patients do not have clinically significant disease, and those with the worst disease often do not present for screening.^30^

There have been multiple efficacy studies for the use of non-mydriatic fundus cameras for AI-based DR screening,^6^^,^^8^^,^^30^ and there are now two US Food and Drug Administration (FDA)-approved devices for autonomous AI-based screening.^31^ Further, there have been a number of exploratory real-world effectiveness studies in low- and middle-income countries.^19^^,^^32^^,^^33^ Nonetheless, translation of this success into real life screening may be a challenge, especially in regions where blindness from DR is increasing, such as in Africa, as it remains to be seen how an AI device (when it has shown effectiveness in the target population and on a device that is available) might be integrated into fragile health systems. Telemedicine has not been widely successful in poorer countries, in part due to the cost of cameras and high-speed data connections. Autonomous AI devices still require trained personnel to operate them, and these people would have to be recruited, trained, and paid. One practical example is that some regions may not have provider capacity to meet the treatment burden generated by screening the entire at-risk population. It is promising that these devices are becoming less expensive and more readily available, as are offline AI solutions. Increased access to cameras will mean greater availability of a diversity of image data from all world regions to further refine AI models, which are often trained with more homogeneous data and which may not perform as well in more diverse populations. Finally, one other barrier to the success of AI implementation for DR screening that must be tackled is patient education. Unfortunately, many patients within the “population at risk” are unaware of their diagnosis or unaware of the need for and importance of screening.^30^ Systems that integrate AI-based screening approaches must be cognizant of the fact that it is often the poorest and least served who will be missed. If the goal of the intervention is to affordably, equitably, and effectively reduce blindness, then we have to focus on all of these factors beyond the performance of an algorithm. In the digital age, this may require out-of-the-box solutions to identify patients who may benefit from screening, raise their awareness of the benefits, and develop systems that are incentivized to maximize population benefit, not just profit.

### Glaucoma

Glaucoma is similar to DR in that it is age related and increasing in prevalence due to population dynamics, it generally has a lengthy and asymptomatic preclinical stage, and there is efficacious treatment that can slow vision loss, essentially preventing functional blindness in a patient's lifetime.^30^^,^^34^ Unlike DR, there continues to be debate as to the best way to diagnose glaucoma and establish gold standards for the development and validation of AI models. Thus, a relatively simplistic model of implementing a population-based screening using non-mydriatic photographs presents a more complex task for glaucoma than for DR.^35^ Moreover, the population at risk is not as specific; thus, implementing an effective screening program has presented the field with a public health challenge.^36^ Finally, treatment depends much more on patient adherence than some other diseases and is prohibitively expensive for patients in many parts of the world, and it is difficult to predict which patients might benefit the most from treatment.^37^

This raises a more existential question about the nature of disease diagnosis in general and the role of AI. Because a key limitation of the training of AI algorithms under the current model is using supervised learning with “gold standard” labels provided, in cases where the gold standard is imperfect we should think beyond AI for image-based diagnosis and focus on AI-based prediction of outcomes.^35^^,^^37^ That is, rather than have the AI tool make a disease *diagnosis* at a point in time, perhaps the better long-term approach is to have the AI tool, in concert with available clinical, demographic, electronic health record, and potentially genetic information, make an outcome *prediction*. AI-based biomarkers, defined based on outcome rather than expert diagnosis could improve the ability to predict structural or functional progression. Moreover, informed by data from multiple randomized trials, AI algorithms that predict disease course could theoretically identify patient-specific IOP treatment goals and even incorporate big data elements such as genomic data.^35^^,^^37^^,^^38^ Fundamentally, considering the role that AI might play in the prevention of glaucomatous vision loss, perhaps even more so than the other diseases described in this paper, reminds us of the myriad roles AI might play in care pathways, beyond image-based detection of disease.

### Age-Related Macular Degeneration

Age-related macular degeneration is the world's most prevalent age-related blinding disorder.^35^^,^^37^^–^^39^ Fueled by an aging population, it has been estimated that the number of people living with AMD globally in 2020 was 196 million, rising to 288 million by 2040.^39^ It is increasingly affecting both high- and low-income countries. A significant pressure point for healthcare systems relates to the high proportion of unnecessary referrals for the vast majority of AMD cases that are non-exudative and for which no treatment is necessary or available, beyond that which the general eye care provider can recommend.^39^^,^^40^ To date, the diagnosis of exudative AMD has required specialist imaging assessments that rely on expensive technologies such as optical coherence tomography (OCT). Of relevance to low- and middle-income countries are two elements: the availability of costly imaging technologies such as OCT and the burden on specialist expertise for the interpretation of imaging tests to exclude neovascular AMD. For the latter, AI offers the potential for automation of the diagnostic process and referral refinement to minimize unnecessary referrals with suspicion of neovascular AMD.^40^ AI decision support systems have recently been developed and shown to have good diagnostic accuracy against human experts in interpreting ocular imaging tests, such as OCT for the diagnosis of urgent maculopathies, such as neovascular AMD.^40^^,^^41^ In theory, systems could be developed that triage the specialist referral process, improving the effectiveness of screening and improving the efficiency of specialty clinics to prioritize those patients who truly need referral. Implementation science can evaluate the cost-effectiveness, patient and practitioner acceptability, usability, and technical integration gap of such AI-enabled referral refinement pathways to allow real-life deployment in healthcare systems.

Such prospective implementation science clinical trial design requires multi-disciplinary expertise from clinicians, health economists, human-computer interaction experts and AI software engineers. Study designs need to be tailored to address the context-specific technical, economic, cultural and logistical barriers to implementation. In the case of low and medium income countries, the wide availability of expensive imaging technologies such as OCT becomes of particular relevance in this use-case.^42^ For those settings, emerging proof-of-concept data for automated AI-enabled neovascular AMD detection based on the interpretation of much more widely available and inexpensive imaging technologies, such as color fundus images, offer potential for driving down costs and increasing the implementation potential of such AI-enabled referral refinement pathways.^40^^,^^41^^,^^43^ Such AI tools still require additional evidence of diagnostic performance from external and prospective validation studies. The training of AI algorithms for detection of neovascular AMD on the basis of color fundus images involves cross-labeling such images using labels derived from OCT interpretation. This use case highlights the transformative potential of AI to enhance the diagnostic performance of more inexpensive technologies that had hitherto been insufficient to support an essential care pathway.

### Retinopathy of Prematurity

Approximately 50,000 babies develop severe visual impairment or blindness annually, many cases of which could be prevented with appropriate screening and timely treatment.^44^ The disease is emerging in many low- and middle-income countries where premature babies are surviving but the number of at risk babies outstrips the ability of the healthcare system to provide adequate retinopathy of prematurity (ROP) screening with indirect ophthalmoscopy.^44^^,^^45^ Telemedicine for ROP has emerged as an alternative to serial physician examinations, and there are a number of successful programs.^44^^–^^46^ In most cases, these have been slow to replicate or difficult to sustain due to the high capital cost of acquiring cameras and the personnel required to take the images. Additionally, these programs are time intensive for physicians who review the images, most of which are normal. Emerging proof-of-concept data in real-world populations indicate that AI-assisted ROP image screening may be effective, such as the i-ROP DL algorithm; however, there are no commercially available products.^47^ Moreover, most of the papers evaluating AI in ROP have used images from a camera that costs more than US$100,000. Future work must be done to validate the efficacy and effectiveness of using lower cost cameras in populations of babies from low- and middle-income countries. Such potential use of AI highlights one additional concept, in that the effectiveness of an AI intervention on the population level depends in large part on factors related to whether the intervention can get to the population at risk. The advantage to ROP is that the entire at-risk population is defined and captive within neonatal care units. In theory, systems of care could be developed that deploy low-cost cameras to every neonatal care unit to provide ROP screening to every at-risk baby in the world.

## Future Directions

In this paper, we have tried to lay out a framework for how to think about the practical steps that must be taken to realize the potential of AI to improve outcomes and reduce preventable blindness. Like many innovative technologies, AI has the potential to widen the technology gap between the rich and the poor if it ends up embedded only in cameras that are prohibitively expensive, employs limited training sets, or is unavailable to the majority of the world's population. On the other hand, unlike many technologies that rely on expensive hardware to implement, AI algorithms could be implemented inexpensively using mobile health technology such as cell phones, and it is not unrealistic to consider that this technology could have the ability to narrow the gap between high- and low-income countries in the provision of services to screen for preventable or treatable blindness.

In order to realize that potential, much more research is needed. We need to develop AI algorithms that work not only with high-quality datasets or images from expensive cameras but also in real-world populations with all of their limitations. We need to encourage innovation in developing AI models that can be used with technologies that are widely available at low cost all around the world. We need to systematically and scientifically evaluate barriers to implementation not just for AI but to many technologies that improve outcomes and demonstrate good value to society. We need funding agencies to recognize the value not just of innovative technology development but also of practical real-world evaluation of existing technologies. AI innovation in medicine has been a success of convergence science, the result of catalytic interactions among multiple domain experts in computer science, engineering, and medicine. AI implementation in medicine will similarly require investment from not only clinicians and technology developers but also key stakeholders invested in health systems, regulatory agencies, and those responsible for healthcare costs and reimbursement. Finally, we need advocacy on behalf of our patients, and regulatory bodies and healthcare payers within each healthcare system should recognize the added value of AI and develop a suitable and profitable environment to encourage not only innovation but also implementation.
